# Atomic High-Spin Cobalt Unlocks Reversible Multi-Electron Transfer Chemistry for Superb Aqueous Zn-Mn Batteries

**DOI:** 10.1007/s40820-026-02328-z

**Published:** 2026-08-10

**Authors:** Yajun Zhao, Qi Li, Yanan Lv, Shuoxiao Zhang, Kai Jiang, Meng Xu, Mudasir Muhammad, Yueyang Wang, Yang Ren, Yi Zhao, Xiaoming Sun

**Affiliations:** 1 State Key Laboratory of Chemical Engineering, College of Chemistry, Beijing University of Chemical Technology, Beijing, 100029 People’s Republic of China; 2 Department of Chemical and Biomolecular Engineering, National University of Singapore, Singapore, 117585 Singapore; 3 Beijing Frontier Research Center On Clean Energy, Institute of Physics, Chinese Academy of Sciences, Beijing, 100190 People’s Republic of China; 4 Department of Physics, City University of Hong Kong, Hong Kong, 999077 People’s Republic of China

**Keywords:** Aqueous Zn battery, Atomic high-spin Co, Reversible MnO_2_ deposition/dissolution, Multi-electron transfer chemistry, High performance

## Abstract

To settle inherent irreversible phase transition and motivate re-dissolution of deposited “dead” MnO_2_ without acid and redox mediator addition, we introduced atomic-dispersed Co atoms with high-spin state into layered MnO_2_, denoted as Co–MnO_2_, via an in situ topological phase transformation strategy, thereby unlocking reversible multi-electron transfer chemistry for superb Zn–Mn batteries. Specifically, atomic-distributed Co atoms within Co–MnO_2_ effectively modulate [MnO_6_] octahedral symmetry and reduce Co–O bond covalency along with enhanced lattice oxygen activity. Based on this, high-spin Co (t_2g_^4^e_g_^2^) greatly mitigates the Jahn–Teller distortion as well as promotes electrolytic MnO_2_ deposited onto the cathode surface completely converted from adsorbed Mn^2+^ for inhibited “Mn dendrites”, achieving reversible MnO_2_/Mn^3+^ and electrolytic MnO_2_/Mn^2+^ reactions with highly thermodynamical favorability. Benefiting from the “two-step, three-electron” mechanism triggered by high-spin Co, Zn//Co–MnO_2_ battery delivers an outstanding capacity of 658 mAh g^–1^ and ultra-long lifespan over 15,000 cycles. This work reveals the critical role of transition-metal spin state modulation for energy-dense and durable Zn-MnO_2_ batteries with reversible multi-electron storage mechanisms.

**Supplementary Information:**

The online version contains supplementary material available at 10.1007/s40820-026-02328-z.

## Highlights

- Atomically dispersed high-spin Co atoms embedded in layered MnO_2_ are realized via in-situ topological phase transformation from CoMn-LDH.
- High-spin Co unlocks fast electrode kinetics in Co-MnO_2_ by regulating the symmetry of [MnO6] and providing available excessive electrons to lattice oxygen.
- The Co-MnO_2_ exhibits dual-energy storage mechanisms of MnO_2_/Mn^3+^ in bulk and electrolytic MnO_2_/Mn^2+^ at interface in Mn^2+^ containing electrolyte.

**Supplementary Information:**

The online version contains supplementary material available at 10.1007/s40820-026-02328-z.

## Graphical abstract

**Figure:**
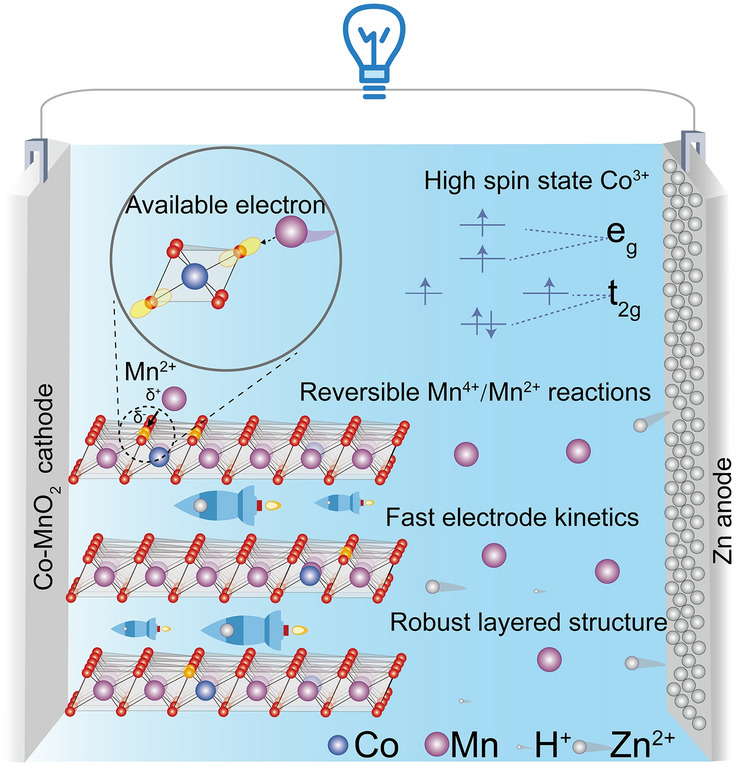

## Introduction

To reduce dependence on fossil fuels and lower carbon emissions, the exploration of renewable energies, such as solar, wind, and tidal energy, from nature has gradually become a global megatrend. However, these intermittent renewable energy sources require advanced energy storage devices to store energy and deliver power through electrochemical reactions [1–3]. The flammability of organic electrolytes and scarcity of Li/Co/Ni resources hinder the employment of commercial lithium-ion batteries in the field of large-scale energy storage [4, 5]. Encouragingly, rechargeable aqueous Zn batteries (AZBs) with high safety and low cost have gained tremendous attention to achieve high-energy and robust batteries based on multi-electron-transfer cathode materials [6–8]. Over the past few decades, manganese dioxide (MnO_2_) has been used as one of the most promising cathode materials for Zn batteries, due to its advantages of outstanding theoretical capacity (308 mAh g^–1^ for Mn^4+^/Mn^3+^) and high redox potential (1.30 V *vs.* Zn^2+^/Zn) compared with vanadium oxides, Prussian blue analogues, organics, and halogen hosts [9–11]. Among various polymorphous MnO_2_ materials (α-, β-, γ-type, etc*.*), δ-MnO_2_ hosts with layered structures are inherently suited for energy storage, as their two-dimensional structure affords favorable ion diffusion pathways [12–17]. However, layered MnO_2_ cathodes typically experience severe Mn dissolution and sluggish reaction kinetics caused by the Jahn–Teller (JT) effect and strong electrostatic interaction between Zn^2+^ and O atoms [18, 19]. Moreover, irreversible products of ZnMn_2_O_4_ and Mn^3+^ disproportionation reaction of “2Mn^3+^  → Mn^2+^  + MnO_2_” further aggravate capacity decay and structural collapse of MnO_2_, thereby resulting in undesirable capacity decay and inferior rate capability [20, 21]. Recently, electrolytic MnO_2_/Mn^3+^ reaction with a high theoretical capacity of 616 mAh g^–1^ and a high voltage of 2.0 V has been reported for cathode-free Zn-MnO_2_ batteries in sulfate-contained electrolytes (pH < 4) [22]. Yet, the strongly acidic electrolytes and the formation of thick electrodeposited MnO_2_ with inferior electronic conductivity often result in severe corrosion in Zn anode and accumulation of electrochemically inactive “dead Mn” owing to incomplete MnO_2_ re-dissolution, thereby limiting the cycling life of Zn batteries. Therefore, Zn//MnO_2_ batteries are still hard to break the bottleneck of capacity and stability merely based on MnO_2_/Mn^3+^ or irreversible MnO_2_/Mn^2+^ reactions within Mn^2+^-additive environments.

Various modifications for MnO_2_ hosts, including surface protection, defect engineering and “guest” pre-intercalation, have been developed to address these challenges by suppressing the JT distortion and Mn^3+^ disproportionation in Mn^2+^-contained electrolytes [23]. However, deposited MnO_2_ from Mn^2+^-containing electrolyte at the cathode/electrolyte interface (CEI) with low reactivity typically induces a transient capacity increase, followed by rapid decay caused by irreversible Mn^2+^/MnO_2_ chemistry [24]. Such abnormal capacity fluctuations mainly originate from non-uniform electrodeposited Mn-species with poor conductivity on the initial MnO_2_ cathode surface, which progressively evolve into electrochemically inactive “dead” MnO_2_ and hinder the subsequent redox reaction of bulk MnO_2_ hosts during prolonged cycling [25, 26]. Previous studies have shown that introducing Co species into Mn-based oxides can enhance structure stability and electronic conductivity by modulating local Mn–O coordination environment, which mitigates JT distortion and improves the cycling stability associated with the MnO_2_/Mn^3+^ redox couple [27, 28]. To accelerate electron transfer kinetics in MnO_2_/Mn^2+^ reaction, redox mediators including I^–^, Fe^2+^, Cr^3+^, V^3+^, and 1,4-benzoquinone have been introduced as “electron bridges” to catalyze the re-dissolution of “dead MnO_2_” into active Mn^2+^, mitigating the capacity decay and enhancing battery reversibility in Mn^2+^ and Zn^2+^ co-existed acidic electrolytes [29, 30]. Besides the negative effect of acidic environment, the inevitable shuttling of these redox mediators might bring new risks of increased self-discharge and Zn corrosion. Beyond that, doping heteroatoms into Mn-based hosts has been proven as an effective strategy to activate MnO_2_/Mn^2+^ redox chemistry in near-neutral Zn batteries without acid addition [31–34]. In particular, doping high-valence transition atoms into the MnO_2_ lattice can regulate the local coordination environment of Mn atoms with introduced electrostatic force, boosting the reversibility of MnO_2_/Mn^2+^ reaction through the acceleration of the Jahn–Teller distortion of Mn^III^O_6_ octahedral for boosted transformation kinetics of primarily determined Mn^IV^ → Mn^III^ step [35]. Very recently, transition metal elements with regulated *d* orbital electronic state can effectively accelerate charge adsorption-transfer kinetics of cathodic hosts, indicating the electrocatalysis synergism motivated by high-spin metal dopants for stable and fast-charging Zn-MnO_2_ batteries [36]. Yet, the irreversible dopant dissolution results in severe capacity decay and limited lifespan of MnO_2_ cathodes during battery cycling, especially in Mn^2+^ containing electrolytes. Moreover, the uniform distribution of dopants within the MnO_2_ lattice structure must be taken into consideration, as non-uniform doping typically gives rise to the heterogeneity and asynchronicity of electrochemical reactions [33]. Mn-based layered double hydroxides (LDHs), such as NiMn-LDH nanoarrays, exhibit a solid solution feature consisting of an ordered Mn^II^ (or Mn^IV^)-O-Mn^III^ structure driven by its heterovalence cation repulsion nature, which provides a desirable frame structure with uniform chemical composition and shortens ion diffusion path for enhanced Zn^2+^ storage [34, 35]. However, the direct application of LDHs as an electrode is not feasible due to strong electrostatic repulsion between terminal H atoms in LDHs and Zn^2+^, leading to inferior capacity and short lifespan.

Herein, we develop a novel Co–MnO_2_ nanoarray cathode featuring atomically dispersed high-spin Co atoms, which is obtained via an in situ electrochemical oxidation transformation of hierarchical CoMn-LDH nanosheets. The incorporation of atomic high-spin Co enables precise structural stability and charge transfer dynamics of MnO_2_ cathode, yielding an impressive electrochemical performance with a maximum capacity (658 mAh g^−1^, 0.2 A g^−1^), superior capacity retention (280 mAh g^−1^, 2 A g^−1^), and ultra-long lifespan over 15,000 cycles (5 A g^−1^). In a combination of experimental and theoretical results, it reveals that atomic high-spin Co with a t_2g_^4^e_g_^2^ electronic configuration can regulate the geometry of [MnO_6_] octahedra, which enhances the intrinsic bulk structural stability of MnO_2_ by suppressing JT distortion. Moreover, the reduced covalency of Co–O bonds renders surface lattice oxygen more available excessive electrons to attract electrolytic carriers (H^+^, Zn^2+^, and Mn^2+^), promoting H^+^/Zn^2+^ diffusion kinetics and re-dissolution of deposited “dead” MnO_2_. As a result, this work establishes the atomic high-spin regulation of transition metal as a typical design principle for advanced Zn batteries with unlocked “three-electron transfer” chemistry of reversible MnO_2_/Mn^3+^ in bulk structure and electrolytic Mn^2+^/MnO_2_ reactions at the cathode interface without acid and redox mediator introduction into electrolytes.

## Experimental Section

### Materials and Reagents

Mn(NO_3_)_2_·4H_2_O, Co(NO_3_)_2_·9H_2_O, MnSO_4_·H_2_O, ZnSO_4_·7H_2_O and KOH were purchased from Sigma-Aldrich Co. Ltd and used without further purification. Deionized (DI) water was used in all the experimental processes. The coin battery products were purchased from Canrd Technology Co. Ltd.

### Synthesis of Co–MnO2 and MnO_2_ Cathodes

CoMn-LDH sample was prepared using a straightforward electrodeposition technique. Carbon cloth (CC) with an area of 3 × 3 cm^2^ was first treated by concentrated nitric acid to form hydrophilic surface. Mn(NO_3_)_2_·6H_2_O and Co(NO_3_)_2_·9H_2_O were then dissolved in 50 mL of DI water, maintaining a total metal ion concentration of 1 mM and a Mn:Co ratio of 20:1, to form the electrolyte for electrodeposition. The CC served as the working electrode, while a saturated calomel electrode and a Pt plate (3 cm width and 3 cm length) were used as the reference and counter electrodes, respectively. The electrodeposition was carried out at − 1.5 V for 600 s to deposit CoMn-LDH onto the CC. Afterward, the CoMn-LDH-coated CC was rinsed with deionized water and dried at 60 °C for 2 h. Mn(OH)_2_ was synthesized using the same process, excluding the addition of Co(NO_3_)_2_·9H_2_O. Electrochemical oxidation processes of CoMn-LDH and Mn(OH)_2_ phase into Co–MnO_2_ and MnO_2_ were conducted by CV tests after 35 cycles at 20 mV s^−1^ within 0 ~ 0.6 V *vs.* saturated calomel electrode in 1 M KOH electrolyte.

### Material and Electrochemical Characterizations

X-ray diffraction (XRD) patterns of MnO_2_ and Co–MnO_2_ were collected using Bruker DAVINCI D8 ADVANCE diffractometer with Cu Kα radiation (λ = 0.15418 nm) at 40 kV, 40 mA. The morphology and elemental composition analysis of MnO_2_, Co–MnO_2_ were obtained by scanning electron microscopy (SEM) with an accelerating voltage of 20 kV and equipped with energy-dispersion spectrum (EDS) mapping equipment (Hitachi Regulus 8230, 10.0 kV) and high-resolution transmission electron microscopy (HR-TEM, TECNAI G2 F30) and the aberration-corrected high-angle annular dark-field scanning transmission electron microscope (HAADF-STEM, JEOL ARM200F, Tokyo, Japan). The valence states of MnO_2_ and Co–MnO_2_ were analyzed by X-ray photoelectron spectroscopy (XPS), the analyses were performed on a Thermo Scientific ESCALAB 250Xi with a mono-chromatized micro-focused Al Ka X-ray source provided by eceshi (www.eceshi.com). Raman spectrometer was carried out with Ar laser excitation source at 532 nm (LabRAM Aramis, HORIBA Jobin Yvon). Inductively coupled plasma atomic emission spectrometry (ICP-AES, Agilent 725ES) was used to measure the Mn ratio for the Co–MnO_2_ at different charged and discharged states.

### Electrochemical Measurements

The CR2032-type coin cells were assembled in air by using MnO_2_ and Co–MnO_2_ as cathodes, 2 M ZnSO_4_ and 0.4 M MnSO_4_ solution as the electrolyte and a Zn foil (99.9% trace metal basis) as the anode separated by a glass fiber separator. Cyclic voltammetry (CV) and electrochemical impedance spectroscopy (EIS) profiles were tested on a CorrTest CS2350H electrochemical workstation (Wuhan CorrTest Instrument Corp., Ltd. China), with a frequency range from 10,000 to 0.01 Hz at 10 mV. The galvanostatic charge–discharge (GCD) experiments of the full zinc ion batteries were carried out using a NEWARE Battery Test System (Neware, CT-ZWJ-4'S-T-1U, Shenzhen, China) at room temperature.

## Results and Discussion

### Design Principle and Structural Characterizations of Co–MnO_2_

**Fig. 1:**
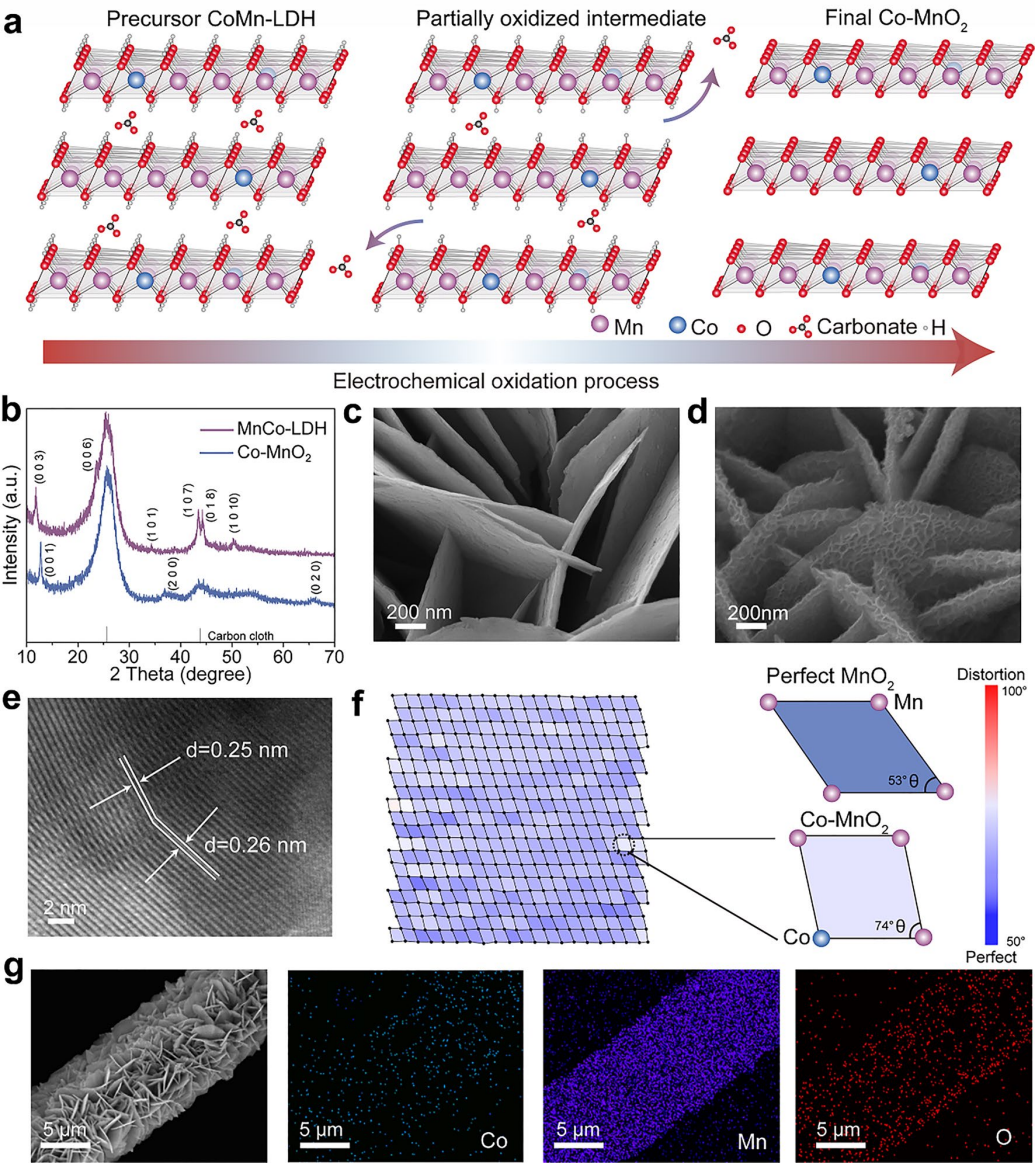
Schematic diagram for the synthesis of Co–MnO_2_ and its corresponding characterization results. **a** Schematic illustration of Co–MnO_2_ along with the removal of interlayered anion guests and Co oxidization. **b** XRD patterns of CoMn-LDH and Co–MnO_2_. SEM images of **c** CoMn-LDH and **d** Co–MnO_2_. **e** TEM image and **f** Theta distribution obtained from HAADF-STEM image of Co–MnO_2_. **g** Elemental mapping images of Co–MnO_2_ with the homogeneous distribution of Co, Mn, and O elements

The atomically dispersed Co within layered MnO_2_ (denoted as Co–MnO_2_) nanosheets was fabricated by in situ electrochemical oxidation of CoMn-LDH in 1 M KOH electrolytes (Fig. S1). As illustrated in Fig. 1a, the CoMn-LDH nanosheets precursor is electrochemically deposited onto the carbon cloth, followed by continuous electrochemical oxidation treatment. During this treatment, the interlayered anion guests, such as carbonate species, were gradually removed, leading to a decreased interlayer distance and accompanied by the oxidization of cations, and eventually achieve the topological transformation from CoMn-LDH to birnessite-type Co–MnO_2_. As shown in X-ray diffraction (XRD) analysis, CoMn-LDH precursor undergoes a topological phase transformation, forming atomically dispersed Co within layered MnO_2_ nanosheets (Fig. 1b). Specifically, CoMn-LDH precursor exhibits characteristic diffraction peaks belonging to (003), (006), and (101) planes of the typical LDH phase with a space group of *R-3 m* (JCPDS card no. 33-0429) [36, 37]. After electrochemical oxidation treatment, Co–MnO_2_ displays a typical birnessite-type MnO_2_ phase (Space group of *C2/m*, JCPDS card No. 43-1456) with typical planes of (001), (002), and (100), indicating a successful phase transformation from CoMn-LDH to layered Co–MnO_2_ [38]. Furthermore, the morphological evolution between CoMn-LDH and Co–MnO_2_ was examined using high-resolution scanning electron microscopy (SEM), as shown in Figs. 1c, d and S2. Different from flower-like CoMn-LDH nanoplatelets, Co–MnO_2_ nanosheets display an evenly vertical-growing nanoarray (lateral length around of 200 nm) with open and porous structures. This unique hierarchical structure offers better wetting behaviors and shorter diffusion paths for carriers, contributing to reduced interfacial resistance and improved rate capability. Moreover, the high-resolution transmission electron microscopy (HR-TEM) images were further conducted to explore the microstructures of Co–MnO_2_ (Fig. 1e). Specifically, a notable lattice fringe with *d*-spacing value of 0.25 ~ 0.26 nm was observed in Co–MnO_2_ nanosheets, which is assigned to (200) plane of δ-MnO_2_ [38, 39]. Besides, the disordered regions (green area) and porous areas (yellow area) were also observed in Co–MnO_2_, which could be attributed to the partial dissolution of Mn cations from the precursor laminates of CoMn-LDH during the in situ oxidation process (Fig. S3a). Furthermore, the Co–MnO_2_ with hierarchical structure exhibits typical diffraction rings for δ-MnO_2_ in the selected-area electron diffraction (SAED) pattern, confirming the successful phase transformation from CoMn-LDH to Co–MnO_2_ (Fig. S3b). Subsequently, aberration-corrected high-angle annular dark-field scanning transmission electron microscopy (HAADF-STEM) was carried out to directly confirm the local atomic environment of Co–MnO_2_. Notably, all transition metals are evenly dispersed within a laminate of layered Co–MnO_2_ (Fig. S3c, d.). Moreover, the variation in the atomic intensity profile recorded along the labeled column indicates Co existed in the form of an isolated single atom. To clarify the influence of Co ion on lattice geometric symmetry of MnO_2_, we characterized the theta (*θ*) for the unit cells corresponding to Fig. 1f, in which *θ* represents the angle between two basis vectors of a unit cell. Compared to *theta* (53°) in the perfect MnO_2_ (*C2/m)*, the wide range of *theta* angle (74°) confirms that the local structure of Co–MnO_2_ deviates considerably from the average cation ordering in perfect MnO_2_. It can be attributed to the lattice distortion of [MnO_6_] octahedron induced by atomically dispersed Co ions in Co–MnO_2_. The corresponding elemental mappings further confirmed the uniform distribution of atomic Co in Co–MnO_2_ (Fig. 1g). Such a structurally homogeneous feature is distinct from conventional ion-doped MnO_2_ cathode, where dopants might be non-uniformly dispersed in MnO_2_ crystal and compositional heterogeneity. In contrast, the hierarchical Co–MnO_2_ nanoarray is derived from a chemically homogeneous CoMn-LDH precursor through in situ electrochemical oxidation process, which enables the uniform incorporation of high-spin Co atoms into the MnO_2_ framework at the atomic scale. Benefiting from this atomic-level distribution, the Co–MnO_2_ nanoarray is expected to deliver improved electrochemical performance for AZBs.

### Electronic and Local Structural Analyses of Co–MnO_2_

**Fig. 2:**
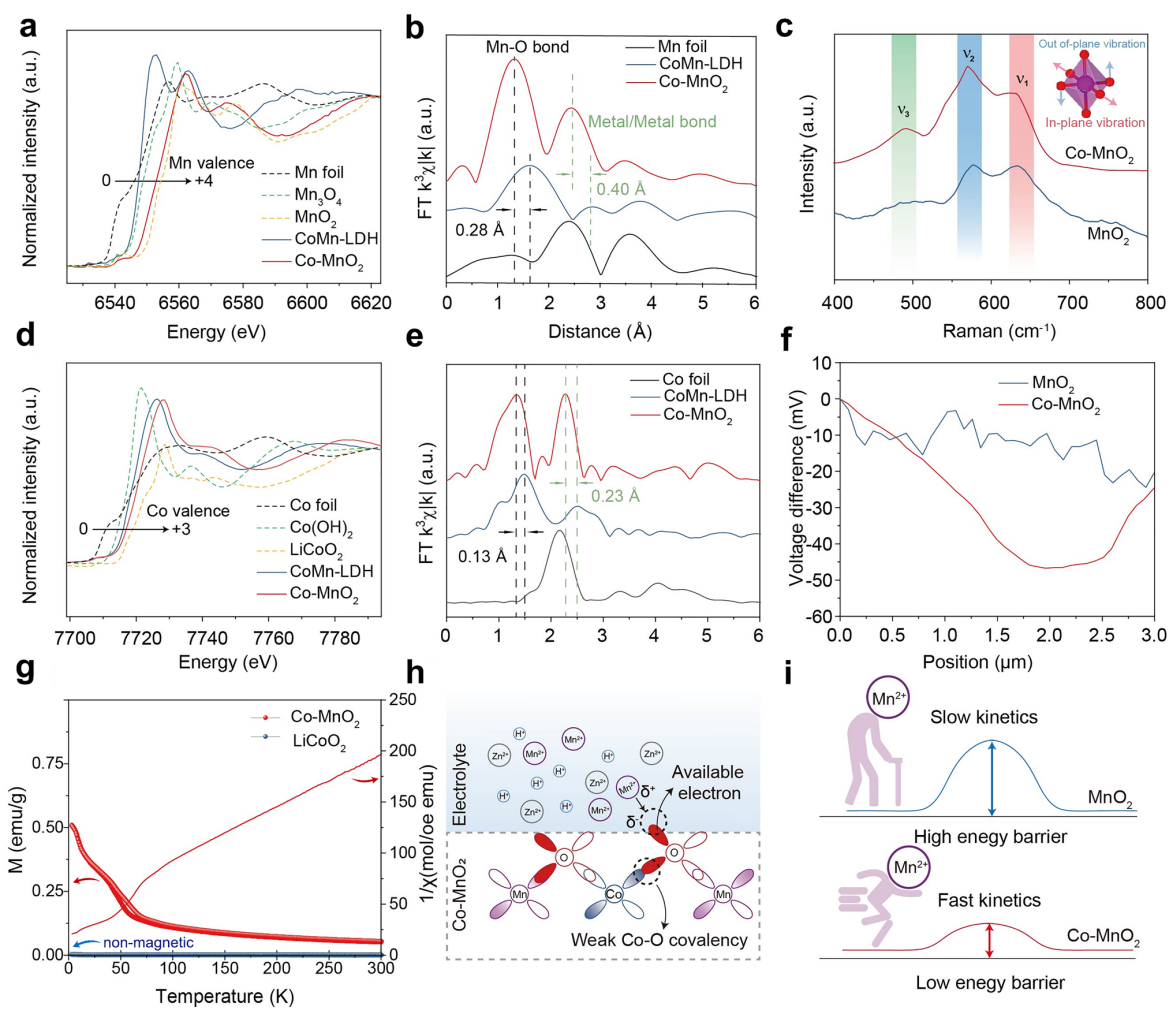
Electronic and local structural characterizations of Co–MnO_2_. **a** Mn K-edge XANES and **b** corresponding FT-EXAFS K^3^χ(R) spectra. **c** Comparison of Raman spectroscopy for MnO_2_ and Co–MnO_2_. **d** Co K-edge XANES and **e** corresponding FT-EXAFS K^3^χ(R) spectra. **f** Built-in potential distribution. **g** Magnetization versus temperature curves of Co–MnO_2_ and LiCoO_2_ reference as well as 1/χ versus temperature with Cuire-Weiss fitting of Co–MnO_2_. **h** Illustration of fast ion transport rates in Co–MnO_2_. **i** Schematic diagram of the slow and fast deposition kinetics of electrolytic Mn^2+^ onto MnO_2_ and Co–MnO_2_

To further investigate the evolution of the coordination environment of transition metals during the phase transformation process, we performed X-ray absorption near-edge spectroscopy (XANES) and extended X-ray absorption fine structure (EXAFS) analysis for CoMn-LDH precursor and Co–MnO_2_. In the Mn K-edge XANES spectra, CoMn-LDH exhibits an absorption edge position lower than that of Mn_3_O_4_, indicating that Mn atoms in CoMn-LDH are predominantly below the + 3 valence state (Fig. 2a). In contrast, the Mn K-edge in Co–MnO_2_ shifts positively to a higher energy position near that of standard MnO_2_, suggesting the successful in situ topological phase transformation of Co–MnO_2_ with increased Mn^4+^ content converted from CoMn-LDH through the electrochemical oxidation treatment. Similarly, Co–MnO_2_ shows a higher Co K-edge than that of CoMn-LDH, indicating an increased valence state for Co atoms featuring a modified orbital electron configuration (Fig. 2d). The Fourier transformed (FT) EXAFS spectra were used to explore the local coordination environment of transition metals in Co–MnO_2_. In Mn K-edge FT-EXAFS k^3^χ(k) spectra, two main peaks attributed to Mn–O and Mn–Mn/Co bonds were shifted from 1.64 and 2.84 Å in CoMn-LDH to 1.36 and 2.44 Å in Co–MnO_2_, respectively (Fig. 2b) [40, 41]. A similar shift was observed in the Co K-edge FT-EXAFS k^3^χ(k) spectra for Co–O and Co–Co/Mn bonds, indicating a different local chemical environment for Mn and Co atoms in CoMn-LDH and Co–MnO_2_ (Fig. 2e). To obtain detailed information about the local coordination environments of transition metals, least-squares EXAFS fittings were conducted. Both Mn–O and Co–O bonds in Co–MnO_2_ exhibit an unsaturated coordination, contributing to the formation of oxygen defects during the phase transformation from CoMn-LDH to Co–MnO_2_ (Table S1). Notably, the coordinatively unsaturated environment in Co–MnO_2_ can trigger the spin transition of Co atoms to a high-spin configuration, affecting the covalency of Co–O bond by reducing the overlap between Co 3*d* orbitals and O 2*p* orbitals. To further confirm the spin state of Co atoms in Co–MnO_2_, the spin configuration is analyzed by the pre-edge peak of Co K-edge spectra associated with Co 1*s*-3*d* transition. In particular, the pre-edge peak intensity of Co in Co–MnO_2_ was higher than that of CoMn-LDH precursor, confirming the presence of high-spin state Co^3+^ induced by its lower degree of central symmetry (Fig. S4) [42]. Besides, Raman spectroscopy of Co–MnO_2_ and MnO_2_ were compared to further investigate the coordination environment of octahedral Mn cations and structural distortions. Compared to layered well-ordered MnO_2_, the peak *ʋ*_1_ intensity of Co–MnO_2_ is reduced owing to its unsaturated coordination environment of Mn/Co–O bonds, confirming the lower [MnO_6_] symmetry induced by atomic Co regulation (Fig. 2c) [11, 43]. To further confirm this, Kelvin probe force microscopy (KPFM) was used to investigate the charge density distribution of Co–MnO_2_ (Figs. 2f and S5). Compared with bare MnO_2_ (0.08 kV cm^−1^), Co–MnO_2_ exhibits a non-uniform potential distribution featuring a higher maximum electric field intensity (0.24 kV cm^−1^) from the surface to the bulk, which is beneficial to the charge transfer process for enhanced rate capability induced by the existing built-in electric field. Moreover, the effective magnetic moments (μ_eff_) are calculated using the temperature-dependent magnetization (M-T) curves for Co–MnO_2_ based on a Curie–Weiss Law (Fig. 2g) [44]. As a result, the fitted total μ_eff_ showed a higher 3.83*μ*_*B*_ than that of mere Mn^4+^ contribution (3.77*μ*_*B*_) in Co–MnO_2_, confirming the presence of approximately 40% atomic ratio of Co^3+^ with high-spin state of t_2g_^4^_eg_^2^ in the Co–MnO_2_ structure. Therefore, high-spin state Co ions render lattice O with available excessive electrons in the cathode surface via an elongated Co–O bond, which facilitates attracting charge carriers at the interface with enhanced electrode kinetics. Moreover, the atomically dispersed Co improves the intrinsic electronic conductivity of MnO_2_, enabling fast electrolytic MnO_2_/Mn^2+^ reaction kinetics at the interface without leading to the formation of “dead MnO_2_” (Fig. 2h, i). Collectively, the high-spin state Co in MnO_2_ can regulate [MnO_6_] local structure and electronic structure, leading to optimized electrode kinetics for high-performance AZBs.

### Electrochemical Performance of Co–MnO_2_ Cathode

**Fig. 3:**
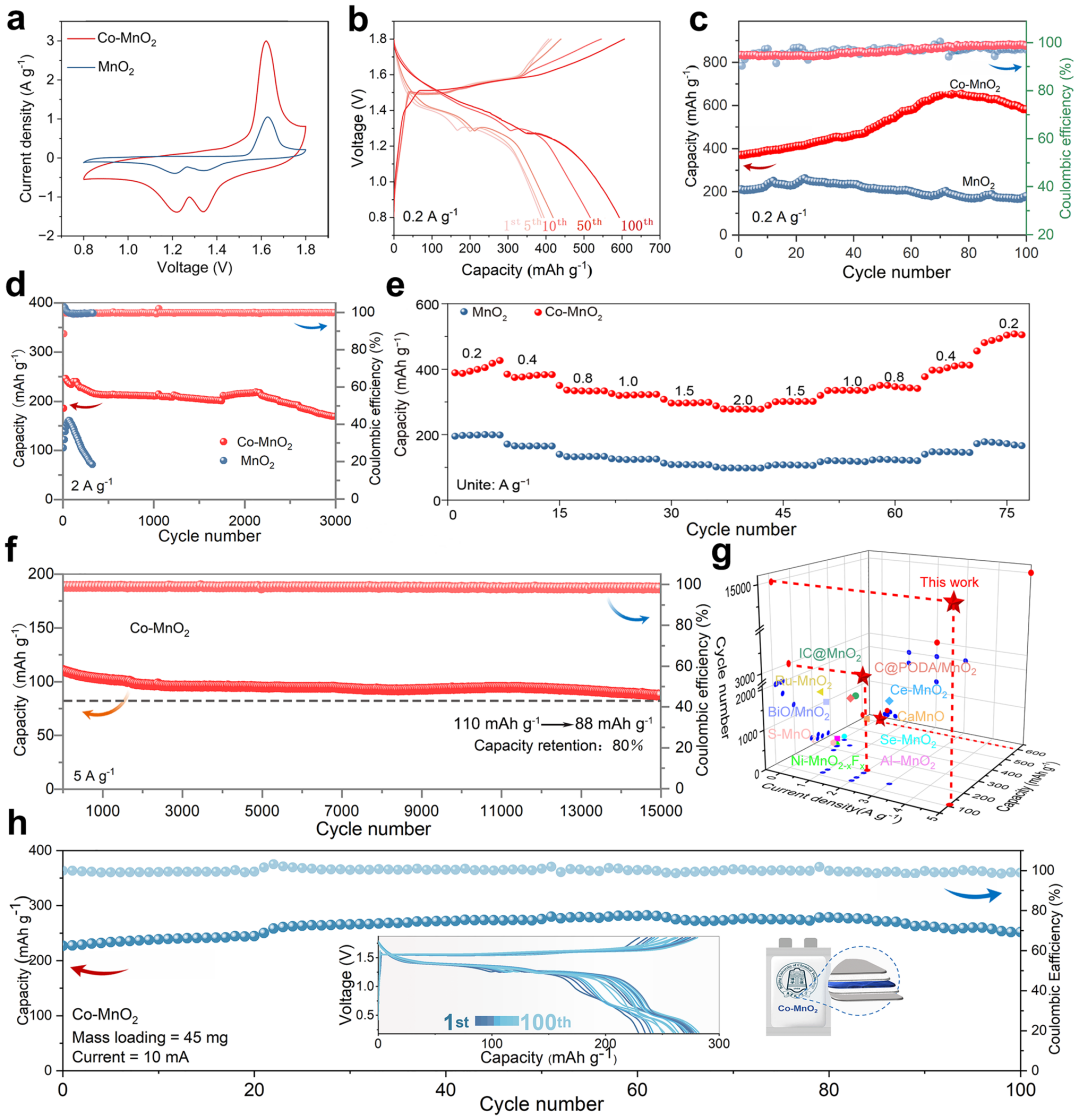
Electrochemical performance of Co–MnO_2_ and MnO_2_ cathodes. **a** CV curves at 0.2 mV s^−1^. **b, c** GCD curves and cycling performance at 0.2 A g^−1^, respectively. **d** Cycling performance at 2 A g^−1^. **e** Rate performance. **f** Cycling performance at 5 A g^−1^. **g** Cycling performance comparison of Co–MnO_2_ with reported Mn-based cathodes. **h** Cycling performance and corresponding GCD profiles of the Zn pouch cell with Co–MnO_2_ cathode after 100 cycles at 10 mA

To analyze the positive effect of atomic-dispersed high-spin Co atoms, a series of Co–MnO_2_ cathodes were prepared by adjusting the mass ratio of Co to Mn (designed as Co_*x*_-MnO_2_) from 1:3, 1:10, 1:20, to 1:30, respectively. According to the comparison of cycling performances, Co_0.05_-MnO_2_ (1:20) was selected as the optimized cathode with the highest capacity and improved stability for the following Zn/Co–MnO_2_ battery fabrication (Figs. S6 and S7). In addition, the cycling performance of Co–MnO_2_ and MnO_2_ cathodes was collected within electrolytes of 2 M ZnSO_4_ and 0.1 ~ 0.5 M MnSO_4_ to identify the optimal Mn^2+^ concentration for achieving high-capacity batteries (Fig. S8). For Co–MnO_2_ cathode, cathodic and anodic peaks at 1.21/1.35 V and 1.62 V can be attributed to the insertion and extraction of H^+^/Zn^2+^ ions during the dual-energy storage process, respectively (Fig. 3a) [45, 46]. Notably, Co–MnO_2_ showed higher current densities of redox peaks than that of bare MnO_2_, suggesting the enhanced reaction activity of Co–MnO_2_ for high-capacity Zn battery. Moreover, Co–MnO_2_ cathode displayed a smaller polarization voltage (0.12 V) than that of 0.2 V in the bare MnO_2_ cathode at 0.2 A g^−1^, indicating enhanced redox kinetics induced by the atomic-level Co ion with high-spin state (Fig. S9). In addition, galvanostatic charge–discharge (GCD) profiles of Co–MnO_2_ were further conducted to reveal the gradually activated MnO_2_/Mn^2+^ chemistry along with the existence of a typical MnO_2_/Mn^3+^ reaction for ultrahigh capacity of Zn//Co–MnO_2_ battery during battery cycling (Fig. 3b). Notably, the GCD evolution of Co–MnO_2_ cathode showed significant changes caused by the improved reaction kinetics throughout the cycling process. Specifically, during charging process, a steep voltage rise over 1.6 V is gradually weakened after 50 cycles. Meanwhile, the voltage plateau around the "turning point" at 1.3 V becomes smoother along with the discharge process, indicating enhanced reaction kinetics for high-rate Zn batteries. More importantly, Co–MnO_2_ cathode initially demonstrates an initial capacity of 400 mAh g^−1^ at 0.2 A g^−1^, which rapidly increases to 510, 658, and finally keeps at 600 mAh g^−1^ after 50, 70, and 100 cycles, respectively. However, MnO_2_ cathode exhibits severe capacity decline after 30 cycles, which demonstrates the intrinsic structural instability of MnO_2_ hosts without Co atom modification (Fig. 3c). These results confirmed that the incorporation of Co ion can effectively activate the reversible MnO_2_/Mn^2+^ reaction originated from the MnSO_4_ additive, thereby achieving the outstanding and stable capacity over 600 mAh g^−1^ after long-term battery working at 0.2 A g^−1^. High‑spin Co^3+^ modulates Mn‑O local structure, suppresses Jahn‑Teller distortion, and activates both bulk intercalation redox and interfacial Mn‑species deposition‑dissolution. Additionally, Co–MnO_2_ cathode demonstrates superior rate capability, with discharge capacities of 430, 390, 350, 315, 300, and 280 mAh g^−1^ at current densities of 0.2, 0.4, 0.8, 1.0, 1.5, and 2.0 A g^−1^, respectively (Fig. 3e). More interestingly, the capacity of Co–MnO_2_ still remains 516 mAh g^−1^ when the current density is reverted to 0.2 A g^−1^ after 78 cycles, proving the high reversibility of MnO_2_/Mn^3+^ and electrolytic MnO_2_ deposition chemistry. Even at high current densities of 2.0 and 5.0 A g^−1^, the Co–MnO_2_ electrode maintains a reversible capacity of 185 and 100 mAh g^−1^ after 3,000 and 15,000 cycles, with a minor capacity decay of 0.43% and 0.0014% per cycle, respectively (Figs. 3d, f and S10). In contrast, the bare MnO_2_ electrode retained only a capacity of 60 mAh g^−1^ and then experienced fast battery failure after 375 cycles. Owing to its enhanced electronic conductivity for the redissolution of deposited “dead” MnO_2_, the layered Co–MnO_2_ cathode with high-spin Co ion exhibits a competitive capacity and rate performance compared to most reported Mn-based cathodes (Fig. 3g, Table S2). These results highlight the enhanced structural stability and boosted reaction kinetics provided by Co atoms with high-spin regulation effect, contributing to superior rate capability and cycling stability of Co–MnO_2_ cathode for high-energy and durable Zn-MnO_2_ batteries. Furthermore, the Zn pouch cell with 45 mg mass loading Co–MnO_2_ cathode displayed ultra-stable cycling performance without capacity decay after 100 cycles, demonstrating its practical application (Figs. 3h and S11). More details of the GCD profiles after different cycles for the pouch cell can be seen in the insert.

### Energy Storage Mechanism of Co–MnO_2_ Cathodes

**Fig. 4:**
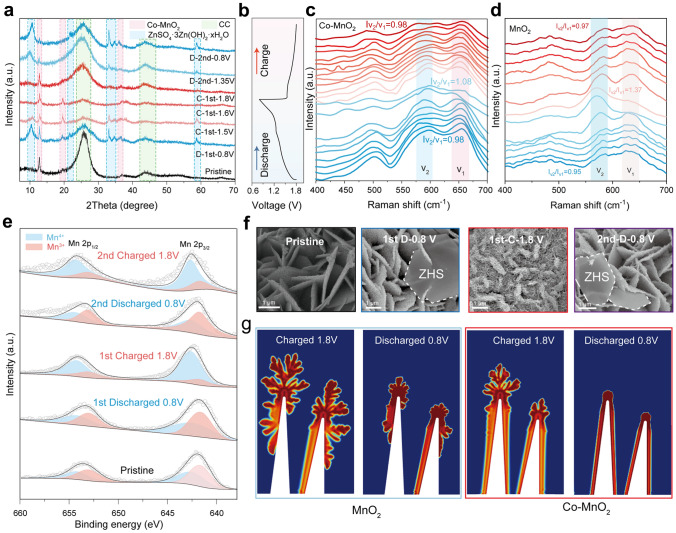
Energy storage mechanism of Co–MnO_2_ cathodes for aqueous Zn batteries. **a** Ex situ XRD, and **b-d** In situ Raman spectra of Co–MnO_2_ and MnO_2_ cathodes at different potentials. **e** ex situ XPS profiles of Co–MnO_2_ during battery cycling. **f** SEM images of Co–MnO_2_ at 0.8 and 1.8 V in the first two cycles. **g** COMSOL Multiphysics simulations of electrolytic MnO_2_/Mn^2+^ reaction on the surface of MnO_2_ and Co–MnO_2_ nanosheets

To better investigate the dual-redox chemistry unlocked by high-spin Co, a series of *in*/ex situ characterizations was conducted on the Co–MnO_2_ cathode during battery working. Figure 4a presents ex-situ XRD patterns at various voltage states to elucidate the robust structural stability of the Co–MnO_2_ cathode, accompanied by cation insertion/extraction. During the discharging process, the characteristic (001) peak located at 12.84°, contributing to Co–MnO_2_, shifts to lower angles (12.72°) and returns to its original position during the following charging, indicating reversible insertion/extraction of H^+^/Zn^2+^ ions [46, 47]. Meanwhile, some new XRD diffraction peaks attributed to Zn_4_(OH)_6_SO_4_·*x*H_2_O (ZHS) (JCPDS Nos. 39–0689, 44–0673, 39–0688) reversibly emerged and disappeared along with the in situ Raman variation at 970–1000 cm^−1^ (SO_4_^2−^) during battery working, further suggesting reversible H^+^ insertion chemistry associated with the ZHS formation/dissolution (Figs. S12 and S13) [48, 49]. Besides, flower-like ZHS with larger flakes form and diminish onto the surface of Co–MnO_2_ electrode during battery discharging and charging processes, respectively, confirming the reversible H^+^ insertion chemistry for high-rate Co–MnO_2_ with high-spin Co atoms (Fig. 4f). Notably, smaller and uniform secondary MnO_2_ nanosheets were appeared and disappeared onto the initial Co–MnO_2_ nanosheets at the 2nd-1.8 V and 2nd-0.6 V, respectively, which is generated by Mn^2+^-dominated reversible MnO_2_/Mn^2+^ reaction from Mn^2+^ containing electrolyte under the regulation of high-spin Co atoms.

In situ Raman spectroscopy was employed to assess the structural stability of Co–MnO_2_ during battery working (Fig. 4b). Three characteristic Raman regions including 640–680 cm^−1^ (*v*_1_ bond), and 572–589 cm^−1^ (*v*_2_ bond), 500–515 cm^−1^ (*v*_3_ bond) are attributed to the symmetric stretching mode and stretching vibrations of Mn–O within the [MnO_6_] octahedra, respectively [50]. Compared with bare MnO_2_, Co–MnO_2_ exhibits reversible shifts of *v*_*1*_ bond between 657 and 651 cm^−1^ during battery cycling, indicating the significantly inhibited Jahn–Teller distortion for improved structural stability of Co–MnO_2_ (Fig. 4c, d). Moreover, the intensity ratio of *v*_*2*_*/v*_*1*_ (I_*v2/v1*_) can serve as an index of the distortion degree of [MnO_6_] octahedra in Co–MnO_2_ hosts [31]. Thus, I_*v2/v1*_ ratio in Co–MnO_2_ cathode was periodically increased from 0.98 (pristine state) to 1.08 (0.8 V) and then decreased to 0.98 (1.8 V) during battery cycling, further confirming the improved cycling stability of the Zn//Co–MnO_2_ battery. In addition, ex situ Mn 2*p* X-ray photoelectron spectroscopy (XPS) peaks of Co–MnO_2_ cathode were collected to verify the reversibility of MnO_2_/Mn^3+^ and electrolytic MnO_2_/Mn^2+^ chemistry during the battery cycling (Fig. 4e). The peaks of Mn 2*p*_3/2_ and Mn 2*p*_1/2_ exhibit a reversible shift with varied intensity for both separated Mn^4+^ and Mn^3+^ peaks, demonstrating the reversible MnO_2_/Mn^3+^ reaction along with Zn^2+^/H^+^ insertion into Co–MnO_2_ cathode. Compared to bare MnO_2_, the enhanced contribution of separated Mn^4+^ peaks in Co–MnO_2_ cathode at 1st- and 2nd-1.8 V further confirms the activated electrolytic MnO_2_ deposition/dissolution chemistry in the Mn^2+^ contained electrolyte triggered by high-spin Co atoms (Fig. S14).

To verify the Mn^2+^-dominated MnO_2_/Mn^2+^ reaction from the electrolyte, inductively coupled plasma emission spectrometry (ICP-OES) was carried out to investigate the evolution of electrolytic Mn concentration at the 1st and 5th cycles in Zn battery with Co–MnO_2_ cathode. Specifically, the electrolytic Mn ion concentration at fully discharged states obviously decreased from 17.864 to 17.376 mg L^−1^ at fully charged 1.8 V after 1st and 5th cycles, respectively (Fig. S15, Table 3.). The electrolytic Mn concentration was increased and kept to a stable value of 18.16 mL^−1^ in the following discharging process. Notably, a larger Mn concentration variation of 18.164 mg L^−1^ (0.8 V) and 17.376 mg L^−1^ (1.8 V) was observed at the 5th cycle, highlighting the role of high-spin Co ion in facilitating reversible electrolytic MnO_2_/Mn^2+^ reaction for high-capacity and stable Co–MnO_2_ cathode. COMSOL Multiphysics simulation model was further constructed to evaluate the effect of high-spin Co ion on reversible MnO_2_ deposition/dissolution behavior onto the cathode surface. Under the same amount of electrolytic MnO_2_ deposition at a fully charged state, bare MnO_2_ nanosheets with poor electronic conductivity exhibits uneven electrodeposited MnO_2_ “dendrites” (Fig. 4g). More seriously, these low-reactive MnO_2_ “dendrites” still existed even at fully discharged state, leading to increased interfacial resistance and electrolytic Mn^2+^ loss for the inferior rate capability and irreversible capacity decay. Due to the high-spin Co modification, Co–MnO_2_ nanosheets with high intrinsic conductivity exhibit relatively uniform deposition and complete re-dissolution of electrolytic MnO_2_ from Mn^2+^-containing electrolyte at 0.8 and 1.8 V, respectively, suggesting the high-reversible MnO_2_/Mn^2+^ chemistry along with MnO_2_/Mn^3+^ reaction. Therefore, the atomic high-spin Co ions can greatly improve structural stability and trigger dual-redox reaction of Co–MnO_2_ cathode for high-capacity and robust Zn batteries without additional additive of acid and redox mediators into electrolytes.

### Theoretical Calculations of Enhanced Dual-Reaction Reversible in Co–MnO_2_

**Fig. 5:**
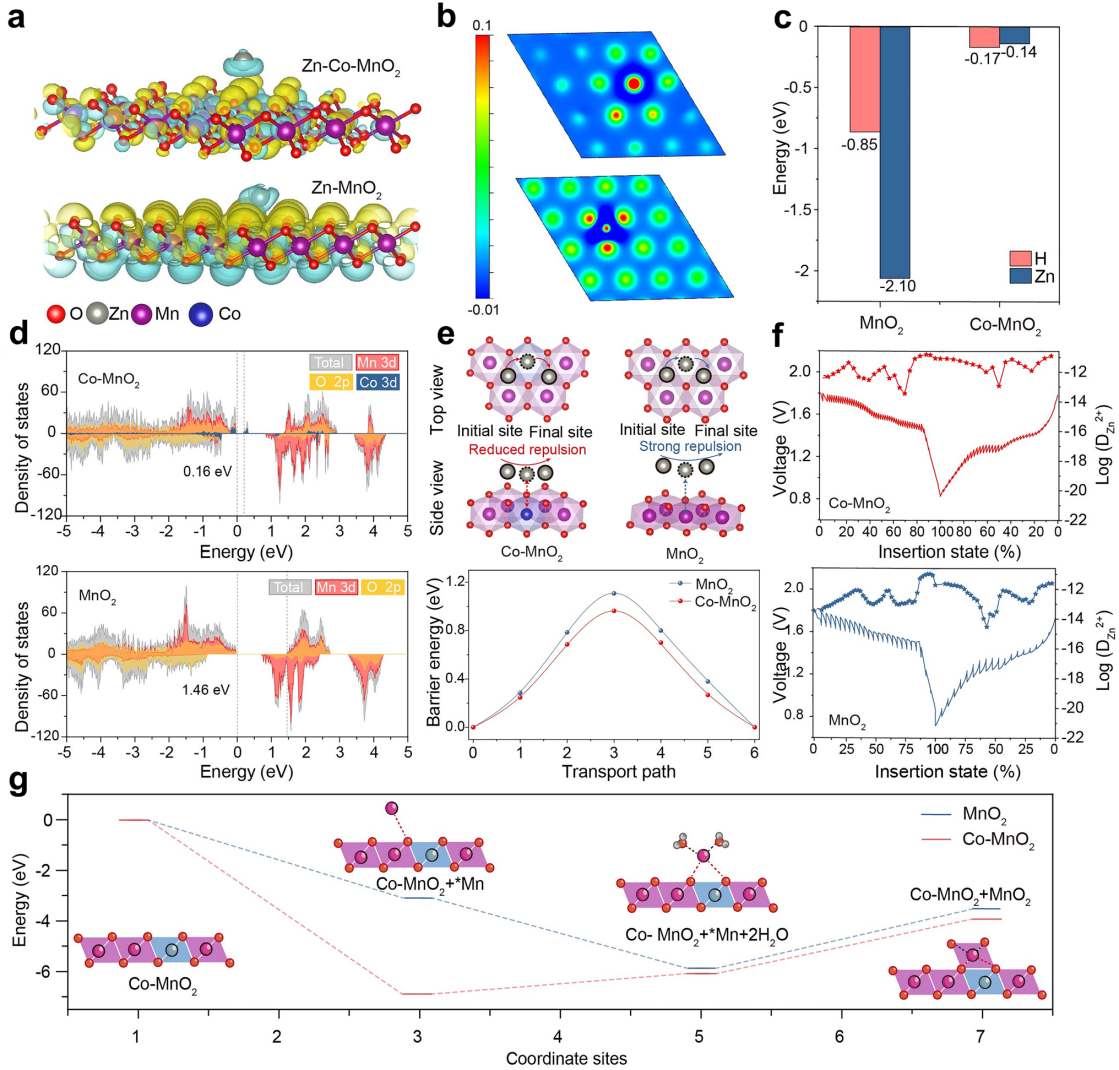
Theoretical calculations of enhanced ion diffusion kinetics and reversible electrolytic MnO_2_/Mn^2+^ in Co–MnO_2_ cathode. **a** Differential charge density with Zn^2+^ insertion in MnO_2_ and Co–MnO_2_. **b** Cross-sectional charge density distributions along ab plane with absorption of Zn^2+^ on the surface of Co–MnO_2_ (top) and MnO_2_ (bottom). **c** Binding energy of H^+^ and Zn^2+^ inserted in MnO_2_ and Co–MnO_2_. **d** Simulated PDOS, **e** schematic illustration and corresponding Zn^2+^ diffusion barriers, and **f** calculated ion diffusion coefficient of MnO_2_ (bottom) and Co–MnO_2_ (top) based on GITT tests. **g** Calculated MnO_2_ deposition energy barriers on MnO_2_ and Co–MnO_2_

Theoretical calculations have been further conducted to elucidate the impact of Co doping on the enhanced electrochemical performance of Co–MnO_2_ cathode. The charge density difference was analyzed to clarify the charge transfer of the inserted ion in both MnO_2_ and Co–MnO_2_, as depicted in Fig. 5a. Notably, bare MnO_2_ exhibited a larger charge depletion area (blue) around the inserted Zn ion, suggesting a stronger electronic interaction between Zn ion and MnO_2_ slab. Conversely, Co–MnO_2_ displayed a smaller charge depletion area, suggesting the Co dopant can effectively mitigate the charge transfer between Zn and the cathode structure, which improves the rate performance of Co–MnO_2_ cathode. Moreover, the corresponding cross-sectional charge density distributions of Zn atom absorbed on the surface MnO_2_ and Co–MnO_2_ along the ab plane (Fig. 5b). It reveals that a high charge density for Zn atom, indicative of a large red area, is obviously observed on the Co–MnO_2_ surface compared to that in bare MnO_2_, confirming that less charge transfer occurs between Zn and Co–MnO_2_. Furthermore, adsorption energies for H and Zn ions were calculated for both MnO_2_ and Co–MnO_2_ cathodes to validate the role of Co dopants. Specifically, bare MnO_2_ cathode exhibits more negative H^+^/Zn^2+^ adsorption energy values of − 2.10/ − 0.85 eV, respectively, than that of − 0.14/ − 0.17 eV in Co–MnO_2_ cathode (Fig. 5c). These results reveal that Co doping effectively weakens the interaction between inserted H^+^/Zn^2+^ and MnO_2_ host, thereby accelerating ion diffusion and reaction kinetics in Co–MnO_2_ cathode.

Additionally, the total density of states (TDOS) and partial density of state (PDOS) for MnO_2_ and Co–MnO_2_ are presented to imply the improved conductivity due to the introduced high-spin Co atoms (Fig. 5d). Specifically, Co–MnO_2_ exhibited a much narrower energy bandgap of 0.16 eV than that of 1.46 eV in MnO_2_ host, which is attributed to the split of e_g_ orbital induced by the high-spin state of Co ions above the Fermi level. As a result, the high-spin state of Co within MnO_2_ slab leads to Co–O bond elongation due to decreased Co–O covalency, which causes a distortion in TMO_6_ octahedral and raises site energy across ion diffusion channels within the interlayer. Introducing chemical coordination distortion will give rise to a wide distribution for the energy of neighboring sites to overlap, which reduces the energy barrier for ion diffusion. In addition, Co–MnO_2_ provides a low electrostatic repulsion pathway for ion migration due to the existence of a lower valence of Co^3+^ compared to Mn^4+^ in perfect MnO_2_. Consequently, Co–MnO_2_ exhibits a lower Zn^2+^ diffusion energy barrier (0.92 eV) compared to MnO_2_ (1.11 eV), indicating enhanced ion (de)insertion dynamics in the Co–MnO_2_ electrode (Fig. 5e). Moreover, the obviously increased ion diffusion coefficient and capacitive contributed capacity in Co–MnO_2_ cathode were observed, which can further confirm the accelerated reaction kinetics in Co–MnO_2_ (Figs. 5f and S16–S19). Electrochemical impedance spectroscopy (EIS) spectra were measured to further investigate the ion diffusion kinetics of the Co–MnO_2_ cathode during the charge/discharge process. As shown in Fig. S20a, the higher charge transfer resistance (R_ct_) represents the slower charge transfer process, which is negative for the fast-charging ability of Zn batteries. Notably, at the fully charged 1.8 V, Co–MnO_2_ with electrodeposited MnO_2_ displays a lower R_ct_ compared with pure MnO_2_, which originates from enhanced electronic conductivity promoted by high-spin Co ations in Co–MnO_2_. After fully discharged to 0.8 V, the R_ct_ for Co–MnO_2_ is relatively increased due to the formation of ZHS with poor electronic conductivity on the cathode surface. Nevertheless, the interfacial impedance of Co–MnO_2_ is still smaller than that of MnO_2_, which confirms the accelerated electrode reactions enabled by high-spin Co ions, verifying that spin state modulation strategy effectively unlocks fast electrode kinetics for aqueous Zn batteries. Moreover, the self-discharge performance of the typical two cathodes was measured by the open circuit voltage tests after 24 h. Specifically, the Co–MnO_2_ cathode exhibits a superior capacity retention of 81.23% after the self-discharge test, outperforming that of pure MnO_2_ with mere 76.02% capacity retention at 3 A g^−1^, which indicates the enhanced cycling stability of Co–MnO_2_ boosted by high-spin Co atoms (Fig. S20b).

To dynamically confirm the H^+^‑involved redox chemistry in Zn‑Mn batteries, in situ pH monitoring of electrolyte was performed during charge/discharge cycling (Fig. S21). For both Co–MnO_2_ and pristine MnO_2_ cathodes, the electrolyte pH presents periodic fluctuations upon charge and discharge processes, demonstrating the reversible H⁺ insertion/extraction involved in the electrochemical reactions. Moreover, a smaller variation of pH values (ΔpH = 0.31) of Co‑MnO_2_ cathode was observed than that of pure MnO_2_ (ΔpH = 0.43), which reveals that high‑spin Co modulation stabilizes the electrode–electrolyte interface by mitigating violent proton‑related interfacial reactions, ensuring the superior cycling stability of Co‑MnO_2_ cathode. Benifiting from the enhanced electronic conductivity and unique ion/electron transport kinetics, Co–MnO_2_ effectively suppresses the formation of electrochemically inactive “dead” MnO_2_ generated by the electrolytic Mn^2+^, leading to an improved the reversibility of dual-energy storage reaction of MnO_2_/Mn^3+^ and MnO_2_/Mn^2+^ redox reactions for Zn//Co–MnO_2_ battery with enhanced capacity and cycling stability.

To better explain the reversibility of the electrolytic MnO_2_/Mn^2+^ reaction on MnO_2_ and Co–MnO_2_ electrode, the DFT calculations on the absorption energy of Mn deposition process were conducted. Figure 5g illustrates the lower absorption energy value (− 6.8 eV) of *Mn on Co–MnO_2_ surface, which is lower than that of MnO_2_ (− 3.1 eV), indicating the Mn^2+^ have thermodynamic preference to absorb on the O atoms near the Co ions in Co–MnO_2_. It could be attributed to the reduced electron localization around lattice O near Co due to the reduced Co–O covalency toward Mn^2+^ adsorption. After electrolytic MnO_2_ formation, the absorption energy (− 3.9 eV) for Co–MnO_2_ is still lower than that of bare MnO_2_ (− 3.6 eV), demonstrating thermodynamically favorable two-electron reaction involved in Mn^2+^-added electrolyte induced by high-spin Co atoms. The incorporation of high-spin Co^3+^ ions (t_2g_^4^_eg_^2^) weakens the covalency of Co–O bonds due to reduced *d–p* orbital overlap, resulting in an accumulation of negative charge around the oxygen atoms in Co–MnO_2_ (Fig. S22). Therefore, an external electric field can be formed within Co–MnO_2_ with the ability to attract excessive positive charge Mn^2+^. Consequently, the Mn^2+^ can be readily adsorbed and lose electrons to oxidized MnO_2_ onto the surface of Co–MnO_2_, thus facilitating the capacity and lifespan of Zn//Co–MnO_2_ batteries.

## Conclusions

In summary, we adopted an electrochemical in situ electrochemical oxidation strategy to successfully realize the transformation from CoMn-LDH precursor to Co–MnO_2_ host with an atomically uniform distribution of Co ions. Notably, the electrooxidation process enables the obtained flower-like Co–MnO_2_ heterostructures with abundant active edges and defective areas, thereby shortening ion diffusion routes for enhanced rate capability. As a result, Co–MnO_2_ cathode exhibits an outstanding reversible capacity of 658 mAh g^−1^, excellent rate capacity, and ultra-long lifespan over 15,000 cycles at 5.0 A g^−1^, offering new approaches to develop a high-spin regulation strategy for advanced Zn-MnO_2_ batteries with multi-electron reactions. More importantly, high-spin Co ion with unsaturated coordination is demonstrated to effectively reduce Co–O covalency to delocalized electrons in O 2*p* orbital, thus attracting more Mn^2+^ to stimulate electrolytic Mn^2+^/MnO_2_ reaction continuously. At the same time, the Co ion can effectively narrow the energy band gap of Co–MnO_2_ with enhanced electronic conductivity, which reduces the formation of “dead MnO_2_” for boosted electrolytic Mn^2+^/MnO_2_ chemistry. This work provides fundamental insights into the role of transition-metal spin state modulation in layered MnO_2_, highlighting its importance for reversible multi-electron storage in advanced Zn–MnO_2_ batteries.

## Supplementary Information

Below is the link to the electronic supplementary material.

Supplementary file1 (DOCX 6668 KB)
