# Supplementary material for: Atomic High-Spin Cobalt Unlocks Reversible Multi-Electron Transfer Chemistry for Superb Aqueous Zn-Mn Batteries
Source: Nanomicro Lett. 2026 Aug 10;19:25. doi: 10.1007/s40820-026-02328-z (PMC13454086; doi:10.1007/s40820-026-02328-z)
Supplement: Supplementary file 1 [file 40820_2026_2328_MOESM1_ESM.docx]

Supporting Information for

**Atomic High-Spin Cobalt Unlocks Reversible Multi-Electron Transfer Chemistry for Superb Aqueous Zn-Mn Batteries**

Yajun Zhao^1,2,3#^, Qi Li^1#^, Yanan Lv^1#^, Shuoxiao Zhang^4^, Kai Jiang^3^, Meng Xu^1^, Mudasir Muhammad^1^, Yueyang Wang^1^, Yang Ren^4^, Yi Zhao^1*^, Xiaoming Sun^1*^

^1^ State Key Laboratory of Chemical Engineering, College of Chemistry, Beijing University of Chemical Technology, Beijing 100029, P. R. China

^2^ Department of Chemical and Biomolecular Engineering, National University of Singapore, Singapore 117585, Singapore

^3^ Beijing Frontier Research Center on Clean Energy, Institute of Physics, Chinese Academy of Sciences, Beijing 100190, P. R. China

^4^ Department of Physics, City University of Hong Kong, Hong Kong 999077, P. R. China

^#^ Yajun Zhao, Qi Li, and Yanan Lv contributed equally to this work.

*Corresponding authors. E-mail: [zybattery@buct.edu.cn](mailto:zybattery@buct.edu.cn) (Yi Zhao); [sunxm@mail.buct.edu.cn](mailto:sunxm@mail.buct.edu.cn) (Xiaoming Sun)

**S1 Calculation Methods**

**S1.1 Theoretical Calculations**

All DFT calculations were performed using the Vienna ab initio Simulation Package (VASP).[S1] The Perdew-Burke-Ernzerhof (PBE) functional within the generalized gradient approximation (GGA) was employed to approximate the exchange and correlation effects of electronic interactions.[S2, S3] The interaction between electrons and ions was described using projector augmented wave (PAW) pseudopotentials.[S4] The DFT+U was applied to treat the strongly correlated cobalt 3d−orbital electrons, with effective parameter of Ueff = U-J for Mn for 4 eV.[S5] The plane wave cut-off energy was set to 450 eV. The cell for MnO_2_ and Co-MnO_2_ model was constructed with 8.5 × 8.5 × 10.2 Å. In structural optimizations, the Brillouin zone was sampled by 3×3×1 k-points using Monkhorst–Pack scheme and energy and force convergence were set at 1.0×10^-5^ eV and 0.01 eV Å^-1^, respectively [S6]. A climbing image-nudged elastic band (CI-NEB) method was used to study the diffusion state using 2×2×2 K-point sampling. Grimme’s DFT-D3 to account for van der Waals interactions, and the dipole correction along the surface normal was considered.[S7]

The binding energies (*E*) of atom (Zn and/or H) on the slab of MnO_2_ and Co-MnO_2_ is determined by calculation:

*E* = *E*_all_–*E*_slab_–*E_atom_*

where E_all_, E_slab_ and E_atom_ represent the total energy with the atoms absorbed on cathode slab, and the energy of the isolated cathode slab in vacuum, the energy of the molecule, respectively.

**S1.2 Diffusion Coefficient**

Galvanostatic intermittent titration technique (GITT) measurements were conducted at a current density of 0.2 A g^−1^, with a galvanostatic pulse time of 300 s followed by a relaxation time of 100 s for each step to achieve steady state equilibrium. The Zn^2+^ diffusion coefficient (D_Zn_^2+^) was calculated based on the simplified Fick’s second law:

$$D=\frac{4}{\pi\tau}{(\frac{mV_{M}}{MS})}^{2}{(\frac{\Delta E_{s}}{\Delta E_{\tau}})}^{2}$$

where τ represents the pulse duration; m, M, and V_M_ are the mass, molar mass, and molar volume of active material, respectively; S is the contact area; ΔE_s_ is the steady state potential variation after relaxation; ΔE_τ_ is the transient potential change during the current pulse.

**S1.3 Effective Magnetic Moment**

The 1/χ versus temperature curve was fitted by Cuire-Weiss law as shown in the following equation:

χ=$\frac{C}{T-\theta}$

where C is Curie constant, *θ* is the Curie-Weiss temperature. As a result, an effective magnetic moment μ_eff_ can be calculated by μ_eff=_$\sqrt{8C}\mu_{B}$_._The calculated μ_eff_ is determined to be 3.83$\mu_{B}$ for Co-MnO_2_, which is higher than μ_eff_ of 3.77 contributed by Mn^4+^ in Co-MnO_2_, thus the extra effective moment is contributed from high spin Co^3+^ (4.90$\mu_{B}$) with t_2g_^4^e_g_^2^ configuration.

**S2 Supplementary Characterizations**

**Fig. S1** Electrochemical oxidation process of CoMn-LDH phase into MnO_2_ phase of (a) Co-doped MnO_2_ and (b) MnO_2_.

**Fig. S2** SEM images at different magnifications of (a, c) CoMn-LDH and (b, d) Co-MnO_2_.

**Fig. S3** (a) HRTEM of Co-MnO_2_. (b) Selected area electron diffraction (SAED) pattern of Co-MnO_2_. (c) Enlarged HAADF-STEM image of Co-MnO_2_ and the corresponding contrast line profile. (d) HAADF STEM images of Co-MnO_2_.

**Fig. S4** The comparison of Co K-edge XANES spectra of CoMn-LDH and Co-MnO_2_.

**Fig. S5** The AFM images of a) CoMn-LDH and b) Co-MnO_2_. Magnetization versus temperature curves.

**Fig. S6** Cycling performance of Co-MnO_2_ cathodes with different Co/Mn ratios.

The electrolytic Mn^2+^ utilization was evaluated based on the electrolyte-driven Mn^2+^/MnO_2_ interfacial conversion, rather than the bulk MnO_2_/Mn^3+^ redox process. To separate these two contributions, the theoretical capacity of the one-electron MnO_2_/Mn^3+^ reaction, 308 mAh g^−1^, was taken as the upper limit of the bulk-phase capacity. Therefore, the capacity exceeding 308 mAh g^−1^ was considered to originate from the electrolytic Mn^2+^/MnO_2_ interfacial reaction. According to the cycling performance at 0.2 A g^−1^, the Co-MnO_2_ cathode delivers capacities of approximately 400, 510, and 658 mAh g^−1^ at the initial, 50^th^, and 70^th^ cycles, respectively. The electrolyte-derived Mn^2+^/MnO_2_ capacities are therefore estimated to be 92, 202, and 350 mAh g^−1^ at the above-mentioned corresponding cycles, giving apparent electrolytic Mn^2+^-derived capacity ratios of 23.0%, 39.6%, and 53.2%, respectively. According to the total addition of 0.4 M MnSO_4_, the electrolytic Mn^2+^ utilization ratio was about 51.1% after 70 cycles for the high-capacity Co-MnO_2_ cathode.

**Fig. S7** Cycling performance of Co-MnO_2_ cathodes with different Co/Mn ratios.

**Fig. S8** Cycling performance of Co_0.05_-MnO_2_ cathodes at varied MnSO_4_ concentrations.

**Fig. S9** GCD profiles of Co-MnO_2_ and MnO_2_ cathodes at 0.2 A g^−1^.

**Fig. S10** GCD profiles of Co-MnO_2_ cathode at (a) 2 A g^−1^, (b) 5 A g^−1^ and (c) various current densities.

**Fig. S11** LED device powered by a Zn//Co-MnO_2_ pouch battery.

The specific-capacity calculation methods for both coin cells and pouch cells are provided, which were calculated from the GCD curves according to **Equation (S1)**:

***C_s_*** = ***I*** × ***Δt*** / ***m***	(S1)

where ***C_s_*** is the specific capacity, ***I*** is the applied current, ***Δt*** is the discharge time, and ***m*** is the mass of active Co-MnO_2_ cathode material. For coin cells, ***m*** refers to the active mass of Co-MnO_2_ loaded on the cathode.

For pouch cells, ***m*** refers to the total active mass of Co-MnO_2_ in the pouch-cell cathode. Specifically, the as-obtained Co-MnO_2_ electrode with an effective area of 3 × 3 cm^2^ and a total active mass of 45 mg was directly used as the pouch-cell cathode. The 11.25 mAh Zn//Co-MnO_2_ pouch cell was assembled with a 70 μm-thick Zn foil anode, corresponding to an areal mass of 50.4 mg cm^−2^, and 2 M ZnSO_4_ + 0.4 M MnSO_4_ electrolyte, followed by vacuum sealing in aluminum laminate packaging. The applied current of 10 mA corresponds to a specific current of 0.22 A g^−1^ based on the Co-MnO_2_ active mass. Accordingly, the pouch cell delivered a high specific capacity of 250 mAh g^−1^, corresponding to a total cell capacity of 11.25 mAh.

Based on the following **Equation (S2)**, the negative/positive (N/P) ratio of the 11.25 mAh Zn//Co-MnO_2_ pouch cell was 33. The Zn anode capacity was estimated based on the theoretical specific capacity (820 mAh g^−1^) of Zn metal, while the measured cathode capacity of 250 mAh g^−1^ was calculated from the active mass of Co-MnO_2_ at 10 mA, as shown in **Fig. 3h**.

R_N/P_ = Q_NE_/Q_PE_ = (820 mAh g^−1^×50.4 mg cm^−2^)/(250 mAh g^−1^×5 mg cm^−2^)=33	(S2)

When normalized by the active mass of Co-MnO_2_, the energy density was calculated to be 325 Wh kg^−1^ based on the measured capacity of 250 mAh g^−1^ and an average voltage of 1.3 V according to Equation (S3).

E_Energy_ = Capacity × Average voltage = (250 mAh g^−1^×1.3 V) = 325 Wh kg^−1^	(S3)

**Fig. S12** XRD patterns of Co-MnO_2_ cathode at fully discharged state.

**Fig. S13** *In-situ* Raman spectra (970~1000 cm^-1^) for the Co-MnO_2_ cathode.

**Fig. S14** *ex-situ* XPS profiles of MnO_2_ cathode during battery cycling.

**Fig. S15** ICP-OES analysis of Mn concentration variation after 1000 times dilution in 2 M ZnSO_4_ + 0.4 M MnSO_4_ electrolyte for Co-MnO_2_ at 1^st^ and 5^th^ cycles.

**Fig. S16** CV curves electrode at different scan rates of (a) MnO_2_ and (c) Co-MnO_2_ electrode. The *b*-values of (b) MnO_2_ and (d) Co-MnO_2_ electrode.

In general, the relationship between the peak current (*i*) and scan rate (*ν*) follows the equation *i = a ν^b^*, where the index *b* reflects a variable that predominantly indicates either a diffusion-controlled process (approximately 0.5) or capacitive-like behavior (approximately 1.0). Compared to bare MnO_2_, the Co-MnO_2_ cathode exhibited higher *b* values of 0.71, 0.81, and 0.59 for the redox peaks of O_1_, R_1_, and R_2_, respectively. These results suggested that cobalt doping significantly enhances reaction kinetics at an atomic level.

**Fig. S17** The scanning rate of Co-MnO_2_ electrode separates the capacitive and diffusion-controlled current contribution at different scan rates.

**Fig. S18** The scanning rate of MnO_2_ electrode separates the capacitive and diffusion-controlled current contribution at different scan rates.

**Fig. S19** Contribution ratios of the capacitive capacities and diffusion-limited capacities at different scan rates in (a) Co-MnO_2_ and (b) MnO_2_ electrodes.

As shown in **Fig. S19**, Co-MnO_2_ cathode demonstrates a higher percentage of capacitive-contributed capacity compared to that of MnO_2_ cathode, confirming the improved ion diffusion kinetics for facilitated rate capability of AZMBs.

**Fig. S20** (a) EIS Nyquist plots of Co-MnO_2_ and MnO_2_ cathodes at fully charged and discharged states. (b) The self-discharge tests of Co-MnO_2_ and MnO_2_ cathodes after 24 hours.

**Fig. S21** *In-situ* pH characterizations of (a) Co-MnO_2_, (b) MnO_2_ in 2 M ZnSO_4_ + 0.4 M MnSO_4_ electrolyte, and the active mass loading are same for both cathodes.

**Fig. S22** The partial charge density of the highest occupied states for Co-MnO_2_. The excessive electron accumulated on one side of O 2p orbital around to Co atom, indicating high electron-donating ability of O atom in Co-MnO_2_.

**Table S1** EXAFS fitting parameters at the Co and Mn K-edge for various samples

| Sample | Path | *N* | R(Å) | σ^2^(10^-3^Å) | ΔE_0_(eV) | R-factor |
| --- | --- | --- | --- | --- | --- | --- |
| CoMn-LDH | Mn-O | 5.8±1.0 | 1.82±0.07 | 6.9±1.3 | -4.04±3.71 | 0.05 |
|  | Co-O | 5.45±0.9 | 1.98±0.09 | 14±1.2 | -3.15±5.13 |  |
| Co-MnO_2_ | Mn-O | 5.5±0.6 | 1.95±0.05 | 9.78±3.2 | -8.5±1.6 | 0.06 |
|  | Co-O | 5.6±1.01 | 1.91±0.01 | 18.60±0.7 | -8.8±4.7 |  |

*N* is the coordination number; *R* is distance between absorber and backscatter atoms; σ^2^ is Debye-Waller factor (represents the thermal and static disorder in absorber-scatterer distances); Δ*E*_0_ is the edge energy shift (the difference between the zero kinetic energy value of the sample and that of the theoretical model). *R* factor is used to assess the goodness of the fitting.

**Table S2** Battery performance comparison of Co-MnO_2_ with reported Mn-based cathodes

| Cathode | Rate performance | Cycling performance |
| --- | --- | --- |
| Ru-MnO_2_ [S1] | 314 mAh g^−1^ at 0.2 A g^−1^  120 mAh g^−1^ at 1.0 A g^−1^ | 83% capacity retention  (2000 cycles at 1.0 A g^−1^ ) |
| Ce-MnO_2_ [S2] | 349 mAh g^−1^ at 0.3 A g^−1^  149 mAh g^−1^ at 3.0 A g^−1^ | 89% capacity retention  (100 cycles at 0.3 A g^−1^ )  100% capacity retention  (2000 cycles at 3.0 A g^−1^ ) |
| Al–MnO_2_ [S3] | 328 mAh g^-1^ at 0.2 A g^−1^ | 98% capacity retention  (200 cycles at 0.2 A g^−1^ ) |
| Ni-MnO_2-x_-F_x_ [S4] | 352 mAh g^-1^ at 0.5 A g^−1^  180 mAh g^-1^ at 2.0 A g^−1^ | 100% capacity retention  (100 cycles at 0.5 A g^−1^ )  89% capacity retention  (1000 cycles at 2.0 A g^−1^ ) |
| S-MnO_2_ [S5] | 324 mAh g^-1^ at 0.2 A g^−1^  150 mAh g^-1^ at 3.0 A g^−1^ | 100% capacity retention  (100 cycles at 0.2 A g^−1^ )  90% capacity retention  (1000 cycles at 3.0 A g^−1^ ) |
| CaMnO [S6] | 290 mAh g^-1^ at 0.3 A g^−1^  140 mAh g^-1^ at 1.5 A g^−1^ | 99% capacity retention  (200 cycles at 0.3 A g^−1^ )  90% capacity retention  (1000 cycles at 1.5 A g^−1^ ) |
| IC@MnO_2_ [S7] | 336 mAh g^-1^ at 0.2 A g^−1^  200 mAh g^-1^ at 2.0 A g^−1^ | 80.3% capacity retention  (1300 cycles at 2.0 A g^−1^ ) |
| C@PODA/MnO_2_ [S8] | 140 mAh g^-1^ at 2.0 A g^−1^ | 89.3% capacity retention  (2000 cycles at 2.0 A g^−1^ ) |
| BiO/MnO_2_ [S9] | 113 mAh g^-1^ at 2.0 A g^−1^ | 100% capacity retention  (2000 cycles at 2.0 A g^−1^ ) |
| MnO_2_@AEPA [S10] | 223 mAh g^-1^ at 0.5 A g^−1^  146 mAh g^-1^ at 1.0 A g^−1^ | 100% capacity retention  (200 cycles at 0.5 A g^−1^ )  97% capacity retention  (1700 cycles at 1.0 A g^−1^ ) |
| Se-MnO_2_ [S11] | 386 mAh g^-1^ at 0.1 A g^−1^ | 100% capacity retention  (100 cycles at 0.1 A g^−1^ ) |
| This work | 658 mAh g^−1^ at 0.2 A g^−1^  180 mAh g^−1^ at 2.0 A g^−1^  100 mAh g^−1^ at 5.0 A g^−1^ | 175% capacity retention  (100 cycles at 0.2 A g^−1^ )  80% capacity retention  (3000 cycles at 2.0 A g^−1^ )  80% capacity retention  (15000 cycles at 5.0 A g^−1^ ) |

**Table S3** ICP results of Mn concentrations in Zn//Co-MnO_2_ batteries at the fully charged and discharged states during battery cycling

| Sample name | Dilution ratio | C (mg/L) | C_0_ (mg/L) |
| --- | --- | --- | --- |
| 1^st^-0.8 V | 1000 | 18.37 | 18,370 |
| 1^st^-1.8 V | 1000 | 17.86 | 17,860 |
| 5^th^-0.8 V | 1000 | 18.16 | 18,160 |
| 5^th^-1.8 V | 1000 | 17.38 | 17,380 |
| 10^th^-0.8 V | 1000 | 19.35 | 19,350 |
| 10^th^-1.8 V | 1000 | 18.64 | 18,640 |

(Test element is Mn, C is instrument reading concentration, Dilution ratio is 1000, C_0_ is the concentration of the original sample solution.)

**Supplementary References**

[S1] S. Wang, S. Yao, F. Zhang, K. Ji, Y. Ji et al., Quantum spin exchange interactions trigger O_p_ band broadening for enhanced aqueous zinc-ion battery performance. Angew. Chem. Int. Ed. **64**(4), e202415997 (2024). <https://doi.org/10.1002/anie.202415997>
[S2] Y. Chen, C. Lin, X. Chen, Z. Lu, K. Zhang et al., Modulating the structure of interlayer/layer matrix on δ-MnO_2_ via cerium doping-engineering toward high-performance aqueous zinc ion batteries. Adv. Energy Mater. **14**(35), 2304303 (2024). <https://doi.org/10.1002/aenm.202304303>
[S3] Y. Zhao, S. Zhang, Y. Zhang, J. Liang, L. Ren et al., Vacancy-rich Al-doped MnO_2_ cathodes break the trade-off between kinetics and stability for high-performance aqueous Zn-ion batteries. Energy Environ. Sci. **17**(3), 1279-1290 (2024). <https://doi.org/10.1039/D3EE01659E>
[S4] J. Lin, Y. Wang, M. Chen, J. Lu, H. Mi et al., Regulating the Gibbs free energy to design aqueous battery-compatible robust host. Adv. Energy Mater., **14**(31), 2401275 (2024). <https://doi.org/10.1002/aenm.202401275>
[S5] Y. Zhao, P. Zhang, J. Liang, X. Xia, L. Ren et al., Uncovering sulfur doping effect in MnO_2_ nanosheets as an efficient cathode for aqueous zinc ion battery. Energy Storage Mater., **47**, 424-433 (2022). <https://doi.org/10.1016/j.ensm.2022.02.030>
[S6] T. Wang, J. Jin, X. Zhao, X. Qu, L. Jiao et al., Unraveling the anionic redox chemistry in aqueous zinc-MnO_2_ batteries. Angew. Chem. Int. Ed., **63**(47), e202412057 (2024). <https://doi.org/10.1002/anie.202412057>
[S7] N. Jiang, Y. Zeng, Q. Yang, P. Lu, K. Qu et al., Deep ion mass transfer addressing the capacity shrink challenge of aqueous Zn-MnO_2_ batteries during the cathode scaleup. Energy Environ. Sci., **17**(22), 8904-8914 (2024). <https://doi.org/10.1039/D4EE02871F>
[S8] Y. Zhao, R. Zhou, Z. Song, X. Zhang, T. Zhang et al., Interfacial designing of MnO_2_ half-wrapped by aromatic polymers for high-performance aqueous zinc-ion batteries. Angew. Chem. Int. Ed. **61(49)**, e202212231 (2022). <https://doi.org/10.1002/anie.202212231>
[S9] X. Zhao, F. Zhang, H. Li, H. Dong, C. Yan et al., Dynamic heterostructure design of MnO_2_ for high-performance aqueous zinc-ion batteries Energy Environ. Sci., **17**(10), 3629-3649 (2024). <https://doi.org/10.1039/D4EE00341A>
[S10] X. Xiao, L. Zhang, W. Xin, M. Yang, Y. Geng et al., Self-assembled layer of organic phosphonic acid enables highly stable MnO_2_ cathode for aqueous zinc batteries. Small, **20**(24), 2309271 (2024). <https://doi.org/10.1002/smll.202309271>
[S11] J. Ye, P. H. Li, Z. Hou, W. Zhang, W. Zhu et al., Se-dopant modulated selective Co-insertion of H^+^ and Zn^2+^ in MnO_2_ for high-capacity and durable aqueous Zn-ion batteries. Angew. Chem. Int. Ed. **63**(43), e202410900 (2024). <https://doi.org/10.1002/anie.202410900>
